# MSC-Exosomes alleviate cognitive impairment after mild traumatic brain injury by inhibiting ferroptosis via PI3K/AKT/mTOR-mediated upregulation of GPX4

**DOI:** 10.1007/s10142-025-01760-5

**Published:** 2025-11-20

**Authors:** Haoyang Hu, Mao Li, Yan Wang, Yang Liu, Hong Zhao, Dengfa Zhao, Pengyu Jiang, Xiaoxuan Yang, Xianyang Chen, Fei Yang

**Affiliations:** 1 Department of Neurology, The First Medical Center, Chinese PLA General Hospital, Beijing, 100853 China; 2 Medical School of Chinese PLA, Beijing, 100853 China; 3 International Department of Affiliated High School of SCNU, Guangzhou, 510635 China; 4 Bao Feng Key Laboratory of Genetics and Metabolism, Beijing, China

**Keywords:** Mild traumatic brain injury, Ferroptosis, Mesenchymal stem cell - derived exosomes, PI3K/AKT/mTOR pathway, Cognitive function

## Abstract

Mild traumatic brain injury (mTBI) is a prevalent condition accounting for over 70% of all traumatic brain injury (TBI) cases, and it is a major cause of posttraumatic cognitive impairment. Ferroptosis, a form of regulated cell death characterized by iron-dependent lipid peroxidation, has been implicated in the pathophysiology of mTBI. However, its precise role in mTBI - induced cognitive dysfunction and potential therapeutic strategies remain unclear. This study aimed to investigate the neuroprotective effects of mesenchymal stem cell - derived exosomes (MSC - Exos) against ferroptosis and cognitive dysfunction following mTBI. We established an mTBI rat model and administered MSC - Exos at different doses. Behavioral assessments, histological and molecular biological analyses, and bioinformatics approaches were used. The results showed that mTBI rats exhibited cognitive impairments, increased lipid peroxidation, and reduced GPX4 expression. MSC - Exos treatment improved cognitive function in a dose - dependent manner, attenuated lipid peroxidation, and restored GPX4 expression. Transcriptomic and bioinformatic analyses revealed that MSC - Exos activated the PI3K/AKT/mTOR signaling pathway, which upregulated GPX4 expression and inhibited ferroptosis. In conclusion, MSC - Exos alleviate cognitive deficits after mTBI by inhibiting ferroptosis via PI3K/AKT/mTOR - mediated upregulation of GPX4, providing a novel therapeutic strategy for mTBI.

## Introduction

Traumatic brain injury (TBI)is a significant global health concern, causing disability among adolescents and adults worldwide. It poses a substantial socioeconomic burden, with an estimated over 50 –60 million new cases each year and direct economic losses exceeding $400 billion (Malhotra et al. 2024; Yan et al. 2024; Giner et al. 2022; Tuominen et al. 2012). In Europe more than 2 million TBI - related hospital admissions occur annually resultingin approximately 82 000 deaths (Majdan et al. 2017; Liu 2025). In the United States, emergency departments handle over 2.8 million TBI cases annually, and about 124,000 individuals develop permanent disability (Centers for Disease Control and Prevention (CDC) 2013). Projectionsindicate that by 2030 TBI will remain among the top three causes of injury - related mortality and long - term disability globally (Maas et al. 2017; GBD 2021).

Mild traumatic brain injury (mTBI), defined by a Glasgow Coma Scale (GCS) score of 13–15, constitutes about 80% − 90% of all TBI cases (España-Irla et al. 2025; Hageman and Nihom 2022). Although most patients recover spontaneouslyin a short time, around 20 % experience persistent neurological dysfunction, including headaches, cognitive impairment, and emotional disturbances (Yuh 2021; Schneider et al. 2022). Cognitive deficits, a crucial pathological feature of mTBI, are often overlooked in clinical practice (Lajeunesse et al. 2019). Even in individuals without obvious symptoms, mTBI can lead to subclinical impairments in information processing speed, attention, and memory (McAllister 2016; Lopez 2025; D 2025; Sanjida et al. 2025). Moreover, mTBI significantly increases the risk of developing neurodegenerative diseases such as Alzheimer’s disease (AD), Parkinson’s disease, and chronic traumatic encephalopathy, especially after repeated injuries (Pszczołowska et al. 2024; Vanderploeg et al. 2005). Emerging evidence suggests that mTBI may exacerbate AD - related neuropathology by promoting β - amyloid (Aβ) deposition, tau hyperphosphorylation, and microglial dysfunction (Zhang et al. 2021; Yamane et al. 2024).

Ferroptosis a form of programmed cell deathinvolving iron - dependent lipid peroxidation, reduced glutathione peroxidase 4 (GPX4) activity, and lipid reactive oxygen species (ROS) accumulation, has become a focus in mTBI research (Xie et al. 2019; Yao et al. 2021). Inhibiting ferroptosis has been shown to alleviate neurological deficits in TBI models (Wang et al. 2022), and ferroptosis inhibitors can reduce neuronal injury and pathological protein deposition in repetitive mTBI models, improving cognitive performance (Zhao et al. 2020). However, research on ferroptosis in mTBI is still limited, and the underlying molecular regulatory network and potential therapeutic targets need further exploration.

Mesenchymal stem cell - derived exosomes (MSC - Exos) have shown promise as a potential treatment option. Compared with traditional stem cell therapy, MSC - Exos have better blood - brain barrier permeability and immunocompatibility while retaining the neuroprotective properties of their parental cells (Terstappen et al. 2021; Song et al. 2021). Recent studies have demonstrated that MSC - Exos can inhibit ferroptosis by regulating mitochondrial autophagy and promoting angiogenesis (Zhang et al. 2023; Wang et al. 2020). However, the exact mechanisms of MSC - Exos in mTBI remain largely unknown.

Therefore, this study aimed to characterize the dynamic changes of ferroptosis after mTBI, evaluate the dose - dependent effects of MSC - Exos on modulating ferroptosis, and explore the molecular mechanisms and therapeutic potential of MSC - Exos in improving cognitive impairment after mTBI.

## Methods

### Experimental animals

A total of 60 male specific pathogen-free (SPF) Sprague-Dawley (SD) rats (6–8 weeks old, weighing 220–240 g) were obtained from the Experimental Animal Center of the Chinese PLA General Hospital (License No. SCXK [Army] 2020–0012). The rats were housed under standard barrier conditions at a controlled temperature of 22 ± 2 °C, 50–60% relative humidity, and a 12 - hour light/dark cycle. Food and water were provided ad libitum. All experimental procedures were approved by the Ethics Committee of the Chinese PLA General Hospital (Approval No. AMC − 2024 − 0315) and conducted in accordance with the ARRIVE guidelines.

### Establishment and evaluation of the mTBI model

A standardized mTBI model was established using a controlled cortical impact (CCI) device (Custom Design USA). Rats were anesthetized with sodium pentobarbital (60 mg/kg intraperitoneally) and placed in a stereotaxic frame (Stoelting USA). The right parietal cortex was exposed via a midline scalp incision (2 mm posterior to bregma and 3 mm lateral to the midline). A corticalimpact was delivered at a velocity of 3 m/s animpact depth of 1 mm, and a dwell time of 3 s. Sham - operated rats underwent the same anesthesia and craniotomy procedures without cortical impact. Postoperative neurological function was assessed daily using the modified neurological severity score (mNSS, 0–18 scale). Only animals with mNSS scores ≤ 6 were included in subsequent experiments.

### Experimental grouping and intervention

Among the 52 mTBI-modeled rats, 46 rats with mNSS ≤ 6 were randomized into four groups: mTBI (saline; *n* = 10), mTBI + MSC-Exos 50 µg (*n* = 12), mTBI + MSC-Exos 100 µg (*n* = 12), and mTBI + MSC-Exos 200 µg (*n* = 12). Sham-operated rats (*n* = 8) received saline.

Human umbilical cord–derived MSC-Exos (Procell Wuhan China) were administeredintraperitoneally at 24 h post-injury. We used 50 100 and 200 µg per rat (by protein mass) chosen from rodent brain-injury literatureindicating 50 –200 µg as a safe and effective range (Otero-Ortega et al. 2020; Zhang et al. 2020; Xiong et al. 2024). These doses were defined as low, intermediate, and high, respectively. The injection volume followed the University of Minnesota RAR guideline (10–20 mL/kg) and was standardized to 5 mL per rat, which remained within the ≤ 20 mL/kg limit for our animals.

### Exosome isolation and characterization

#### Exosome isolation

Human umbilical cord-derived mesenchymal stem cells (hUCB-MSCs) were culturedin serum-free, exosome-depleted DMEM for 48 h. Exosomes wereisolated from the conditioned medium using differential centrifugation followed by ultracentrifugation. First, cells and debris were removed by centrifuging at 300 × g for 10 min, 2000 × g for 30 min, and 10,000 × g for 45 min. The supernatant was then filtered through a 0.45 μm PES membrane and ultracentrifuged at 100,000 × g for 2 h. The exosome pellet was resuspended in ice-cold PBS, ultracentrifuged again to enhance purity, and stored at −80 °C. Protein concentration was determined using the BCA assay.

#### Morphological characterization by transmission electron microscopy (TEM)

The morphology of MSC-Exos was examined using TEM. A 20 µL aliquot of the exosome suspension was adsorbed onto a copper grid and negatively stained with 2% uranyl acetate for 10 min. The grid was air-dried and examined using a JEM-1400Plus transmission electron microscope (JEOL). Images were captured at magnifications of 10,000–50,000×. Exosomes were identified by their typical cup-shaped or spherical vesicular structure.

#### Size distribution and concentration analysis by nanoparticle tracking analysis (NTA)

Exosome size distribution and concentration were measured using a ZetaView PMX 110 instrument (Particle Metrix). The exosome suspension was diluted with filtered PBS to achieve a concentration of 1 × 10⁸ to 1 × 10⁹ particles/mL. The sample was measured at 11 positions within the cell, and the size range, particle concentration. All analyses were performed in triplicate.

#### Identification of Exosomal markers by Western blotting

Exosome proteins were extracted using RIPA buffer and quantified by BCA assay. Equal amounts of protein were separated by SDS-PAGE, transferred to PVDF membranes, and blocked with 5% non-fat milk. Membranes were incubated overnight with primary antibodies against exosomal markers CD9(Proteintech Group, Wuhan, China, 20597-1-A), CD63Proteintech Group, Wuhan, China, 25682-1-AP, CD81Proteintech Group, Wuhan, China, 27855-1-AP) and the negative control Calnexin(Abcam, Cambridge, UK, ab22585). After washing, membranes were incubated with HRP-conjugated secondary antibodies. Protein bands were visualized using enhanced chemiluminescence, and band intensities were quantified with ImageJ software. The presence of CD9, CD63 and CD81 confirmed exosome identity, while the absence of Calnexin indicated no contamination from endoplasmic reticulum-derived vesicles.

### Behavioral assessments

All behavioral tests took place in a dedicated room. A video-tracking system recorded every training and test session.

For Novel Object Recognition (NOR). Each rat first habituated to an open-field box for 10 min. In the familiarization session we placed twoidentical objects symmetrically in the arena. Rat explored freely. A dual-channel stopwatch recorded time at each object. The session ended when total exploration reached 20 s or when 10 min elapsed. Six hours later we ran the test session. We replaced one familiar object with a novel object that differedin shape and texture. The volume was similar to avoid size bias. Rat explored for 10 min. We recorded exploration time for each object and calculated the discrimination index (DI).

For Morris Water Maze (MWM).The pool had a 105-cm diameter. Water temperature was 22 ± 1 °C. We added non-toxic white tempera paint (10 mL/L) to make the water opaque. A transparent acrylic platform (10 cm diameter) was submerged 1.5 cm below the surfacein the goal quadrant. Four distinct wall cues provided spatial references. For spatial acquisition, we released each rat facing the wall from pseudo-random start points. Each trial lasted up to 90 s. Each rat completed four trials per day with a 30-mininter-trial interval for four days. We recorded escape latency as the time from water entry to reaching the platform. For the probe trial, we removed the platform. We released rat from the quadrant opposite the former goal. They swam for 60 s. We calculated the percentage of time in the original goal quadrant to assess spatial memory.

### Histological and molecular biological analyses

#### Hematoxylin and Eosin (H&E) staining

Hippocampal tissues were fixedin 4 % paraformaldehyde embeddedin paraffin, and sectioned at a thickness of 4 μm. Sections were stained with H&E and observed under a light microscope (BX53, Olympus, Japan).

#### Detection of ferroptosis markers

Malondialdehyde (MDA) levels were measured using a commercial ELISA kit (Beyotime Institute of Biotechnology, Shanghai, China, S0131). 4 - hydroxynonenal (4 - HNE) levels were assessed using an ELISA kit (FineTest, Wuhan, China, ER1587).

#### Immunohistochemistry (IHC)

Paraffin - embedded sections were subjected to antigen retrieval and sequentially incubated with the primary antibody GPX4 (1:200, Abcam, Cambridge, UK, ab125066). Sections were then incubated with HRP - conjugated secondary antibodies (1:500, Proteintech Group, Wuhan, China, RGAR001), developed with 3,3’ - diaminobenzidine (DAB) substrate, and imaged using a light microscope. The percentage of positively stained cells was quantified using ImageJ software.

#### Western blotting

Total protein was extracted from hippocampal tissues using RIPA lysis buffer. Protein samples were separated by SDS - PAGE and transferred onto polyvinylidene fluoride (PVDF) membranes. After blocking with 5% nonfat milk, the membranes were incubated overnight with primary antibodies against PI3K(1:1000, Cell Signaling Technology, Danvers, MA, USA,4257), P - PI3K (1:1000, Cell Signaling Technology, 4228), AKT(1:1000, Cell Signaling Technology,4691), P - AKT (1:1000, Cell Signaling Technology, 4060), mTOR(1:1000, Cell Signaling Technology,2972), P - mTOR (1:1000, Cell Signaling Technology,2971), and GAPDH(1:1000, Cell Signaling Technology,5174). After washing, the membranes were incubated with HRP - conjugated secondary antibodies (1:2000, abcam, ab205718) for 1 h. Protein bands were detected using enhanced chemiluminescence༈ECL༉regent (Bio-Rad, Hercules, CA, USA,1705061), and the band intensities were quantified using ImageJ software. Band intensities of phosphorylated proteins were normalized to their respective total proteins (p-PI3K/PI3K, p-AKT/AKT, p-mTOR/mTOR).

### Transcriptomic sequencing and bioinformatics analysis

#### RNA extraction and sequencing

Total RNA was extracted from hippocampal tissues using trtizol reagent (Thermo Fisher Scientific Shanghai China 15596026) according to the manufacturer’sinstructions. RNA integrity was assessed using an Agilent 2100 Bioanalyzer (Agilent Technologies, USA). After constructing the cDNA libraries, sequencing was performed on the Illumina NovaSeq 6000 platform (Illumina, USA) to generate paired - end reads of 150 bp (PE150).

#### Data processing

Raw sequencing reads were quality - controlled using FastQC (v0.11.9, Babraham Bioinformatics, UK) and aligned to the Rattus norvegicus reference genome (Rnor_6.0) using HISAT2 (v2.2.1). Differential gene expression analysis was conducted using DESeq2 (v1.38.0), with thresholds set at |log₂ fold change| ≥ 1 and an adjusted p - value < 0.05.

#### Functional enrichment and network analysis

We contextualized the DEG set defined above (|log₂FC| ≥ 1, FDR < 0.05). We performed KEGG over-representation analysis with clusterProfiler v4.6.0 and visualized the top pathways with enrichplot v1.20.0; significance was set at FDR < 0.05. We also ran Gene Set Enrichment Analysis (GSEA) in clusterProfiler v4.6.0 using MSigDB v7.5.1 (Hallmark collection and KEGG C2). For each gene set we report ES, NES, nominal p, and FDR q; we considered FDR q < 0.25 significant.

For Weighted Gene Co-expression Network Analysis (WGCNA; v1.70-3), we used variance-stabilized expression values. We constructed networks with soft-threshold power (β) = 6, minimum module size = 30, and module-merging height = 0.25. We screened outliers by hierarchical clustering and PCA, and removed samples with connectivity Z.k < − 2.5. Hub genes were defined as |MM| ≥ 0.80 and |GS| ≥ 0.30.

We generated protein–protein interaction (PPI) networks in STRING v11.5 and visualized them in Cytoscape v3.9.1.We ranked nodes using degree betweenness and closeness centrality to identify pathway-relevant hubs. We validated selected hubs (Akt mTOR) by qRT-PCR using Gapdh as theinternal control and 2 ^−ΔΔCt for quantification.

### Cellular experiments

#### Cell culture and experimental grouping

Rat hippocampal HT22 cells were cultured in high-glucose DMEM (Gibco Thermo Fisher Scientific Waltham MA USA 11965092) with 10% FBS and 1% penicillin-streptomycin at 37 °Cin a 5 % CO₂ humidifiedincubator. Cells were passaged every 2 –3 days at 80–85% confluence, using passages 3–8 for experiments to ensure consistent phenotype and viability.

Ferroptosis wasinduced by treating cells with Erastin for 24 h (10 µM; Beyotime Institute of Biotechnology Shanghai China SC0224). MSC-derived exosomes were added 1 h before Erastin at 1 µg/µL based on dose-response experiments. To inhibit the PI3K/AKT/mTOR pathway the following inhibitors were used: PI3K inhibitor: LY294002 (20 µM; Beyotime S1737) AKT inhibitor: MK-2206 (5 µM; Beyotime SF2712) mTOR inhibitor: Rapamycin (10 µM; Beyotime S1842)Inhibitors were dissolvedin DMSO and applied 30 min prior to Erastin treatment, with a final DMSO concentration < 0.1%. Cells were randomly assigned to five experimental groups (*n* = 5 per group): (1) Ferroptosis model (Erastin only), (2) MSC-Exos + PI3K inhibitor + ferroptosis model, (3) MSC-Exos + AKT inhibitor + ferroptosis model, (4) MSC-Exos + mTOR inhibitor + ferroptosis model，and (5) MSC-Exos + ferroptosis model.

#### Detection of labile iron pool (LIP)

LIP levels were measured using the Calcein-AM/Fe²⁺ quenching assay (Invitrogen Thermo Fisher Scientific Waltham MA USA C3099) following the manufacturer’s protocol. HT22 cells were seededin 96 -well black-walled plates (Corning Corning NY USA 3904) at a density of 5 × 10³ cells per well. After treatment the cells wereincubated with 2 µM Calcein-AMin serum-free DMEM for 30 min at 37 °C. Fluorescence intensity was measured using a microplate reader.

#### qPCR for ferroptosis-associated genes

For qPCR the amplification was done using the TB Green Premix Ex Taq II Kit on a StepOnePlus Real-Time PCR System. The reaction mixture (20 µL) contained 10 µL of TB Green Premix 0.4 µL of forward and reverse primers (10 µM) 2 µL of diluted cDNA and 7.2 µL of nuclease-free water. The amplification protocolincluded an initial denaturation at 95 °C for 30 s, followed by 40 cycles of 95 °C for 5 s and 60 °C for 30 s. A melting curve analysis at 90 °C was performed to verify the specificity of the PCR products.

#### TEM for mitochondrial ultrastructure

After treatment cells were trypsinized centrifuged at 800 × g for 5 min and fixed with 2.5% glutaraldehydein 0 .1 M PBS at 4 °C overnight. Cells were washed three times with PBS post-fixed with 1% osmium tetroxide for 1 h and rewashed with PBS. The cells were dehydrated using a graded ethanol series and embeddedin Epon 812 resin. Ultrathin sectionswere cut using an ultramicrotome, mounted on copper grids, and stained with 2% uranyl acetate for 15 min and lead citrate for 10 min. Sections were observed with a JEM-1400Plus transmission electron microscope (JEOL) at 80 kV. Images were captured with a Gatan Orius SC1000 camera. Five random fields per group were analyzed to assess mitochondrial morphology, including swelling, cristae disruption, and matrix electron density.

#### Flow cytometry analysis of lipid ROS

Lipid ROS levels were detected using the C11-BODIPY 581/591 probe (Invitrogen Thermo Fisher Scientific D3861). After treatment the cells wereincubated with 5 µM C11-BODIPY 581/591in serum-free DMEM for 30 min at 37 °Cin the dark. The cells were then trypsinized, washed twice with cold PBS, and resuspended in 300 µL PBS. Flow cytometry was performed on a BD FACSCanto II flow cytometer (BD Biosciences, USA), collecting 1 × 10⁴ events per sample. Excitation was set at 488 nm, with emission detected at 515 nm (green fluorescence, oxidized C11-BODIPY) and 585 nm (red fluorescence, reduced C11-BODIPY). The green-to-red fluorescence ratio was used to quantify lipid ROS levels. Data were analyzed with FlowJo software.

#### Cell viability assay

Cell viability was evaluated using the Cell Counting Kit-8 (CCK-8 Beyotime Institute of Biotechnology China) according to the manufacturer’sinstructions. After the indicated treatments, 10 µL of CCK-8 solution was added to each well of a 96-well plate andincubated for 1 –2 h at 37 °C. Absorbance was measured at 450 nm using a microplate reader, and results were expressed as relative viability normalized to the control group.

#### ELISA for ferroptosis-related markers

The levels of MDA, 4-HNE, and GPX4 in cell lysates were quantified using commercial ELISA kits (Beyotime Institute of Biotechnology, China), following the manufacturer’s protocols. Data were normalized to total protein concentration, which was determined by bicinchoninic acid (BCA) assay.

### Statistical analysis

All statistical analyses were performedin GraphPad Prism 9 .4. Data are reported as mean ± SD. Two-group comparisons used unpaired, two-tailed Student’s *t* tests. Multiple-group comparisons used one-way ANOVA with Bonferroni’s post hoc correction. Repeated-measures data used two-way repeated-measures ANOVA with Dunnett’s multiple comparisons test. Exact two-tailed p values are reported in the text or figure panels together with significance asterisks; **ns** denotes not significant. (Optional) Effect sizes (Cohen’s *d* or η²) are provided where appropriate. Unless otherwise specified, data are presented as mean ± SD. Exact n values are indicated in figure legends. All tests were two-tailed.

## Results

### Characterization of mTBI model and MSC-Exos

Sixty rats were usedin this study. Eight rats were assigned to the sham group. The other fifty-two rats underwent mTBI modeling. Forty-six rats had mNSS between 0 and 6. These rats wereincluded in the study. Six rats had mNSS scores higher than 6 and were excluded from further experiments.

**Fig. 1:**
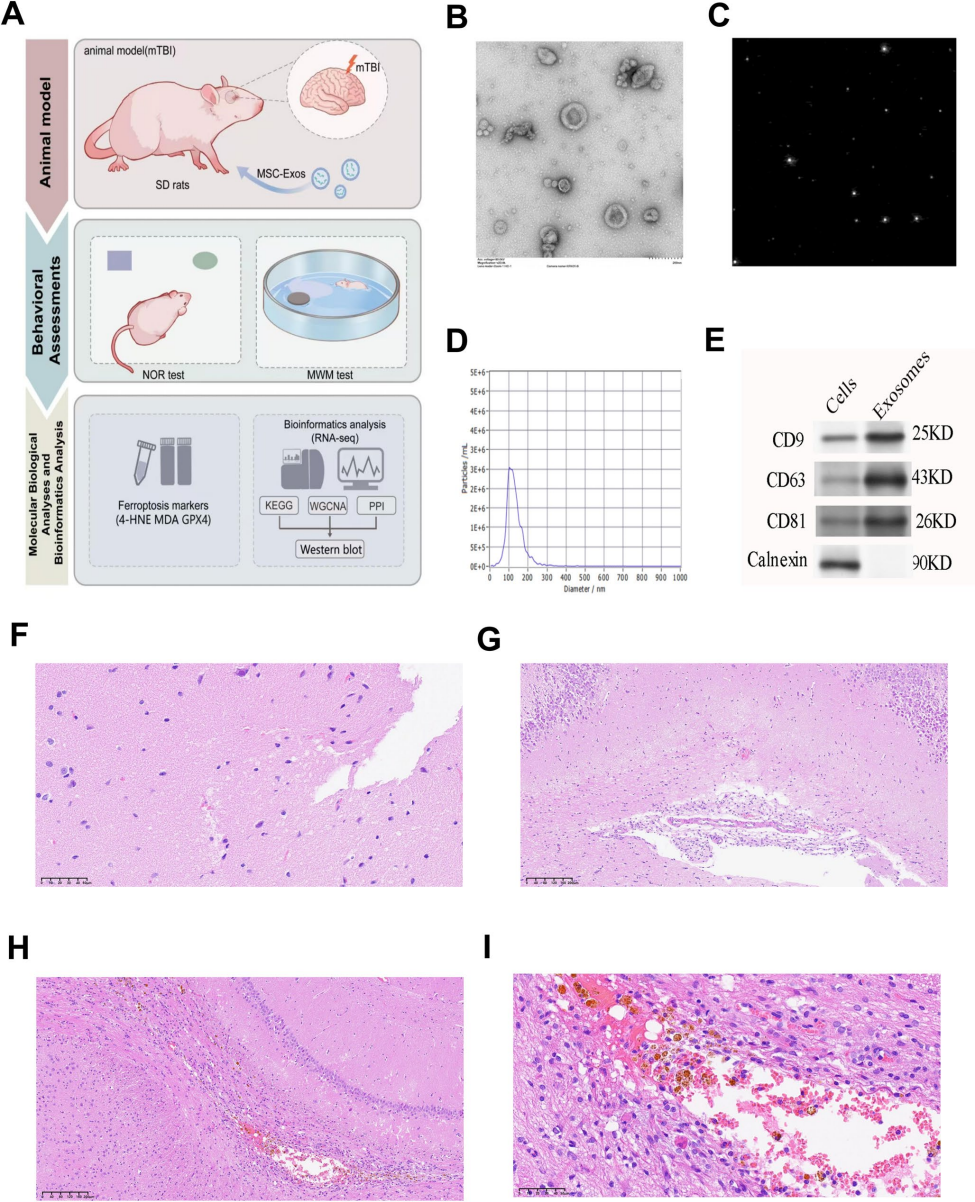
Characterization of MSC-Exos and histopathological validation of the mTBI rat model. **A** The diagram shows the overall experimental design. Rats with mTBI received intraperitoneal injections of MSC-Exos. Behavioral performance was tested using the NOR and MWM tasks. Molecular studies measured ferroptosis markers and gene expression changes. **B–E** These panels confirm the identity of MSC-Exos. (B) TEM shows their typical round or cup-shaped form with clear lipid membranes. (C) NTA reveals the distribution of exosomes in suspension. (D) The size distribution peaks between 80 and 120 nm, which matches the expected range for exosomes. (E) Western blotting confirms specific exosomal markers (CD9, CD63, CD81) and the absence of Calnexin, an endoplasmic reticulum protein. **F**–**I **These panels show the histological features of the mTBI model. (F–G) H&E staining of sham-operated rats shows normal hippocampal structure, with neurons arranged neatly and no signs of inflammation. (H–I) In contrast, rats excluded from the mTBI group (mNSS > 6) show severe tissue damage, including neuronal necrosis, disrupted architecture, and hemorrhage

TEM showed that MSC-Exos had a round or cup-shaped structure with smooth membranes. NTA showed most particles ranged from 80 to 120 nm in size. Western blotting confirmed that MSC-Exos expressed CD9, CD63, and CD81 but not Calnexin (Fig. 1B–E). These results confirmed that the isolated MSC-Exos were pure and correctly identified.H&E staining showed mild edema and slightly disorganized neurons in the hippocampus of mTBI rats. The cortical structure remained intact with no bleeding or cavity formation (Fig. 1F–G). Rats with mNSS scores above 6 showed clear neuronal damage, tissue disorganization, and bleeding (Fig. 1H–I). These findings are consistent with previously reported characteristics of mTBI (Zhang et al. 2023; Omelchenko et al. 2019; Arneson et al. 2022).

### Intervention with MSC-Exos improves cognitive function

#### MSC-Exos treatment enhances spatial learning and memory in mTBI rats

To evaluate spatial learning and memory, rats were subjected to the MWM test, which consisted of navigation (acquisition) and probe trials. In the navigation trials, rats in the mTBI group exhibited significantly longer escape latencies compared with those in the sham group (*p* < 0.05), indicating impaired spatial learning. MSC-Exos treatment reduced escape latency in a dose-dependent manner:

- 50 µg: 51 ± 9 s (*p* < 0.05 vs. mTBI).
- 100 µg: 43 ± 7 s (*p* < 0.01 vs. mTBI).
- 200 µg: 32 ± 4 s (*p* < 0.001 vs. mTBI).

In the spatial probe trial, mTBI rats spent significantly less time in the target quadrant (19.2 ± 2.4 s vs. 25.6 ± 2.1 s, *p* < 0.01) and had fewer platform crossings (1.7 ± 0.4 vs. 3.1 ± 0.5, *p* < 0.01) than sham rats, reflecting impaired memory retention.

**Fig. 2:**
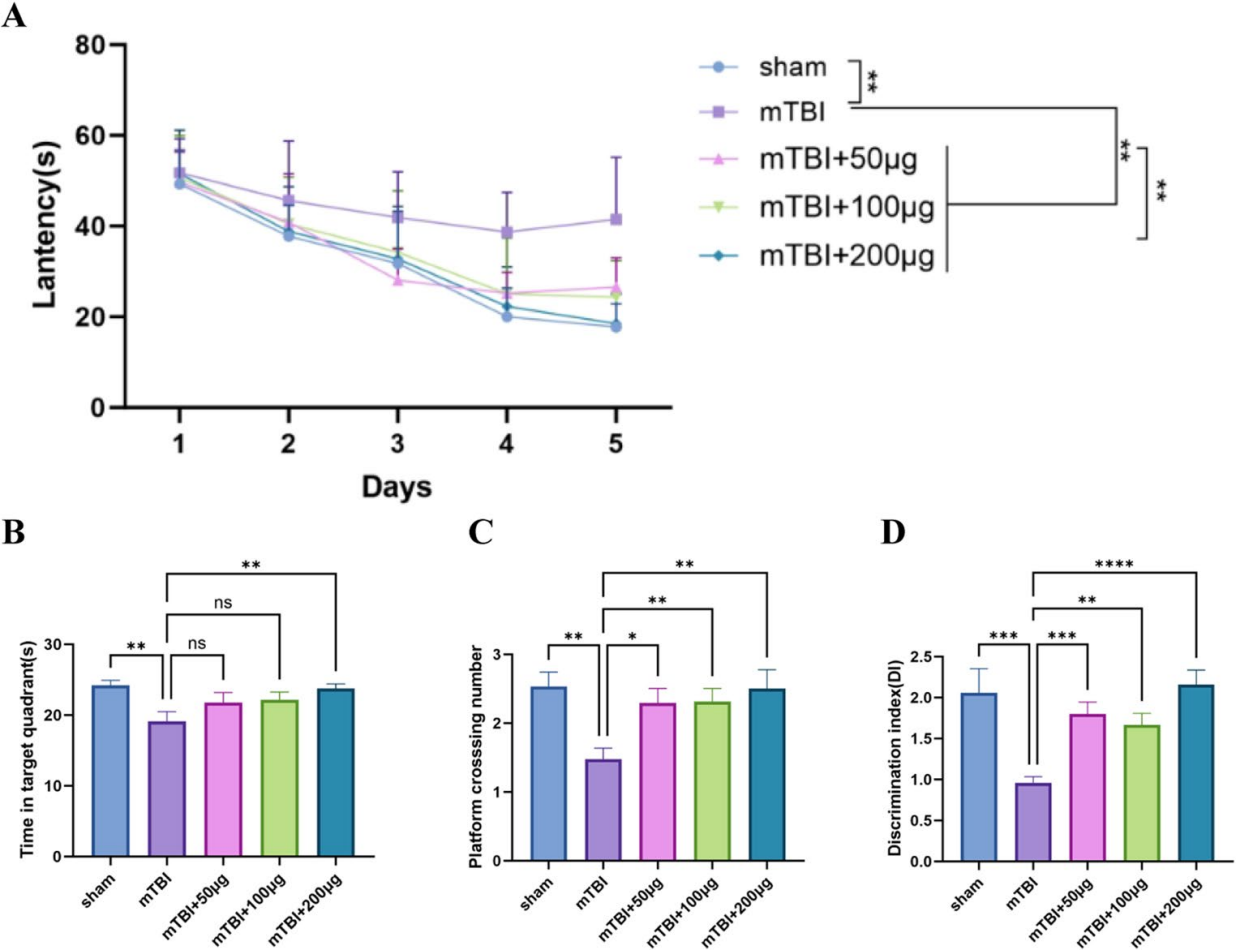
MSC-Exos improve cognitive deficits in mTBI rats. **A **Escape latency over the 5-day training period in the MWM navigation test, reflecting spatial learning ability. **B** Time spent in the target quadrant during the probe trial, used to assess spatial memory retention. **C** Number of platform crossings reflecting spatial memory performance. **D** Discrimination index (DI) in the NOR test, used to evaluate recognition memory. Data are presented as mean ± SD. Two-tailed tests were used. One-way ANOVA with Bonferroni’s post hoc test or two-way RM-ANOVA with Dunnett’s multiple comparisons, as appropriate. n per group: Sham = 8; mTBI = 10; MSC-Exos 50 µg = 12; 100 µg = 12; 200 µg = 12.*p < 0.05, **p < 0.01, ***p < 0.001, ****p < 0.0001; ns: not significant

MSC-Exos treatment significantlyimproved both parameters, with a dose-dependent increase in the time spent in the target quadrant and the number of platform crossings. In the 200 µg group rats spent 24.7 ± 2.2 sin the target quadrant and had 3 .5 ± 0.6 times platform crossing, values comparable to those of the sham group (*p* > 0.05), indicating near-complete recovery of spatial memory function(Fig. 2).

#### MSC-Exos treatment improves novel object recognition in mTBI rats

In the NOR test, rats in the mTBI group exhibited significantly lower DI values than those in the sham group (1.02 ± 0.25 vs. 2.13 ± 0.32, *p* < 0.001), indicating impaired recognition memory. MSC-Exos treatment significantly increased DI values in a dose-dependent manner:

- 50 µg: 1.85 ± 0.27 (*p* < 0.001 vs. mTBI).
- 100 µg: 1.90 ± 0.30 (*p* < 0.001 vs. mTBI).
- 200 µg: 2.41 ± 0.28 (*p* < 0.0001 vs. mTBI; *p* < 0.05 vs. lower-dose groups).

These resultsindicate that MSC-Exos treatment effectively alleviated recognition memory deficits in mTBI rats, with the 200 µg dose showing the most pronounced therapeutic benefit(Fig. 2).

### Transcriptomic and molecular validation of the PI3K/AKT/mTOR pathway in mTBI and its modulation by MSC-Exos

RNA sequencingidentified 3 713 DEGs using thresholds of |log₂FC| ≥ 1 and FDR < 0.05. KEGG pathway analysis showed that these genes were enriched in ferroptosis, PI3K-AKT signaling, and mTOR signaling pathways.

**Fig. 3:**
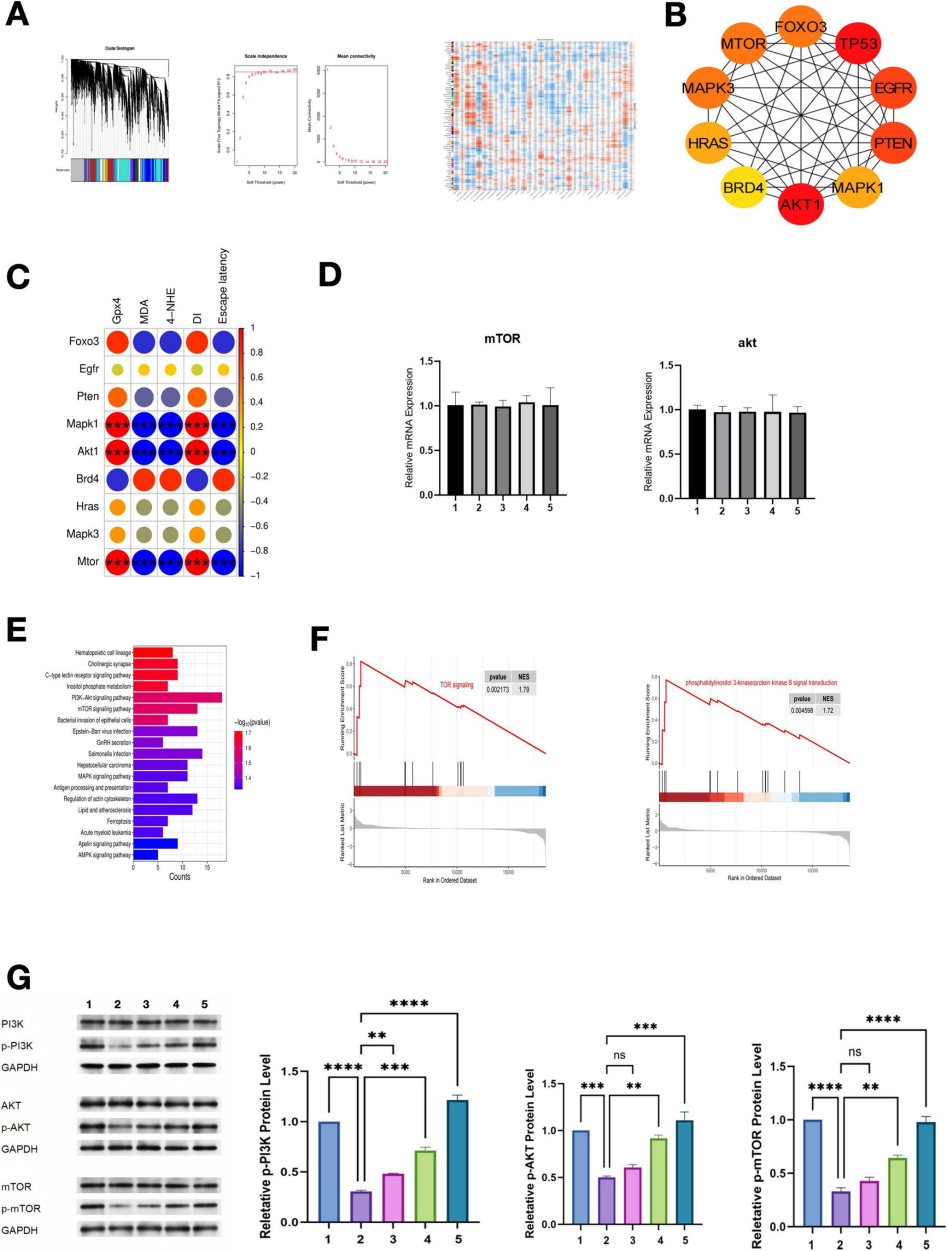
Transcriptomic and molecular validation of the PI3K/AKT/mTOR pathway in mTBI and its modulation by MSC-Exos. **A** This figure shows the transcriptomic data of hippocampal tissue. WGCNA grouped genes into color modules based on expression patterns. The panels show a gene expression density plot, a PCA plot, and a heatmap of DEGs in the Sham, mTBI, and MSC-Exos-treated groups. The data show that mTBI changes many genes, and MSC-Exos help bring them closer to normal. **B** This panel shows the protein–protein interaction (PPI) network of DEGs in the PI3K/AKT/mTOR pathway. Each node is a protein, and each edge is a connection. **C** This heatmap shows the links between PI3K/AKT/mTOR-related genes and markers of ferroptosis and cognitive function. **D** This panel shows quantitative RT-PCR results for mTOR and Akt mRNA in the hippocampus. **E** This KEGG pathway chart shows which biological pathways change in mTBI. The PI3K/AKT/mTOR pathway is one of the most affected ones, based on the number of genes and the − log₁₀(p-value). **F** This GSEA compares the PI3K/AKT/mTOR pathway between groups. The left plot shows that the pathway is lower in mTBI rats. The right plot shows that it goes up again after MSC-Exos treatment. The enrichment scores and p-values are given for both. **G** This panel shows western blot and densitometric data for PI3K, AKT, mTOR, and their phosphorylated forms. GAPDH is the control. Data are presented as mean ± SD (*n* = 6 per group). Two-tailed tests were used. One-way ANOVA with Bonferroni’s post hoc correction (*p < 0.05, **p < 0.01, ***p < 0.001, ***p < 0.0001; ns = not significant). Group information: (1) Sham group, (2) mTBI group, (3) mTBI + MSC-Exos 50 µg group, (4) mTBI + MSC-Exos 100 µg group, (5) mTBI + MSC-Exos 200 µg group

Weighted gene co-expression network analysis (WGCNA) was performed to find gene clusters related to cognitive function after mTBI. Twelve co-expression modules were identified (Fig. 3A). Among them, the blue module had the strongest positive link with cognitive performance (*r* = 0.78, *p* < 0.001).This module contained 342 genes mainly involved in ferroptosis and mTOR signaling. Protein–protein interaction (PPI) network analysis identified several key genes in the blue module (Fig. 3B). These included GPX4, which regulates ferroptosis, and major proteins in the PI3K/AKT/mTOR pathway, such as AKT1 and mTOR. The expression of these hub genes was strongly related to ferroptosis markers (GPX4, MDA, 4-HNE) and behavioral indicators of cognitive function (discrimination index and escape latency) (Fig. 3C).Quantitative real-time PCR (qRT-PCR) was performed to measure the mRNA levels of mTOR and Akt (Fig. 3D). The results showed no significant differences among the five groups. The expression of both genes stayed steady in the sham, mTBI, and MSC-Exos-treated groups. These findings suggest that MSC-Exos did not change the transcription of mTOR or Akt. The activation of the PI3K/AKT/mTOR pathway likely occurs mainly through post-translational regulation.

KEGG pathway enrichment analysis was used to explore the biological functions of the differentially expressed genes (DEGs) (Fig. 3E). The results showed that these genes were enriched in several signaling pathways, including PI3K–AKT, mTOR, ferroptosis, and MAPK pathways. The PI3K–AKT and mTOR pathways showed the highest enrichment scores. These pathways are known to regulate neuronal survival, oxidative stress, and energy metabolism. Other enriched pathways included lipid and atherosclerosis, regulation of actin cytoskeleton, and hematopoietic cell lineage. These pathways suggest that mTBI may also influence cytoskeletal structure and immune activity. Overall, the enrichment pattern supports the idea that MSC-Exos protect neurons and reduce ferroptosis through activation of the PI3K/AKT/mTOR pathway. GSEA confirmed that the PI3K/AKT and mTOR signaling pathways were upregulated in the MSC-Exos-treated group compared with the mTBI group (Fig. 3F). The normalized enrichment scores (NES) for these pathways were 1.72 and 1.79, both statistically significant (*p* < 0.05, FDR q < 0.25).

Western blot analysis further tested the activation of this pathway (Fig. 3G). In mTBI rats, phosphorylated PI3K, AKT, and mTOR were much lower than in the sham group (*p*< 0.01) showing that the pathway was suppressed after injury. MSC-Exos treatment restored phosphorylation in a dose-dependent manner.p-PI3K levels dropped to 0.48 ± 0.06in mTBI rats compared with 1 .28 ± 0.08 in the sham group (*p*< 0.0001). They rose to 0.58 ± 0.05 0.89 ± 0.07 and 1.32 ± 0.06 after MSC-Exos treatment at 50 100 and 200 µg respectively. p-AKT alsoincreased from 0 .45 ± 0.05in the mTBI group to 0 .67 ± 0.06 and 0.79 ± 0.05in the 100 and 200 µg groups (***p < 0.0001). p-mTOR levels showed a similar pattern, rising from 0.44 ± 0.05 to 1.06 ± 0.06 with higher MSC-Exos doses.

These results show that MSC-Exos activate the PI3K/AKT/mTOR signaling pathway at the protein phosphorylation level, mTOR/Akt transcripts remained unchanged across groups. This activation may help reduce ferroptosis and improve neural recovery after mTBI.

### MSC-Exos inhibit ferroptosis progression

#### Lipid peroxidation levels

**Fig. 4:**
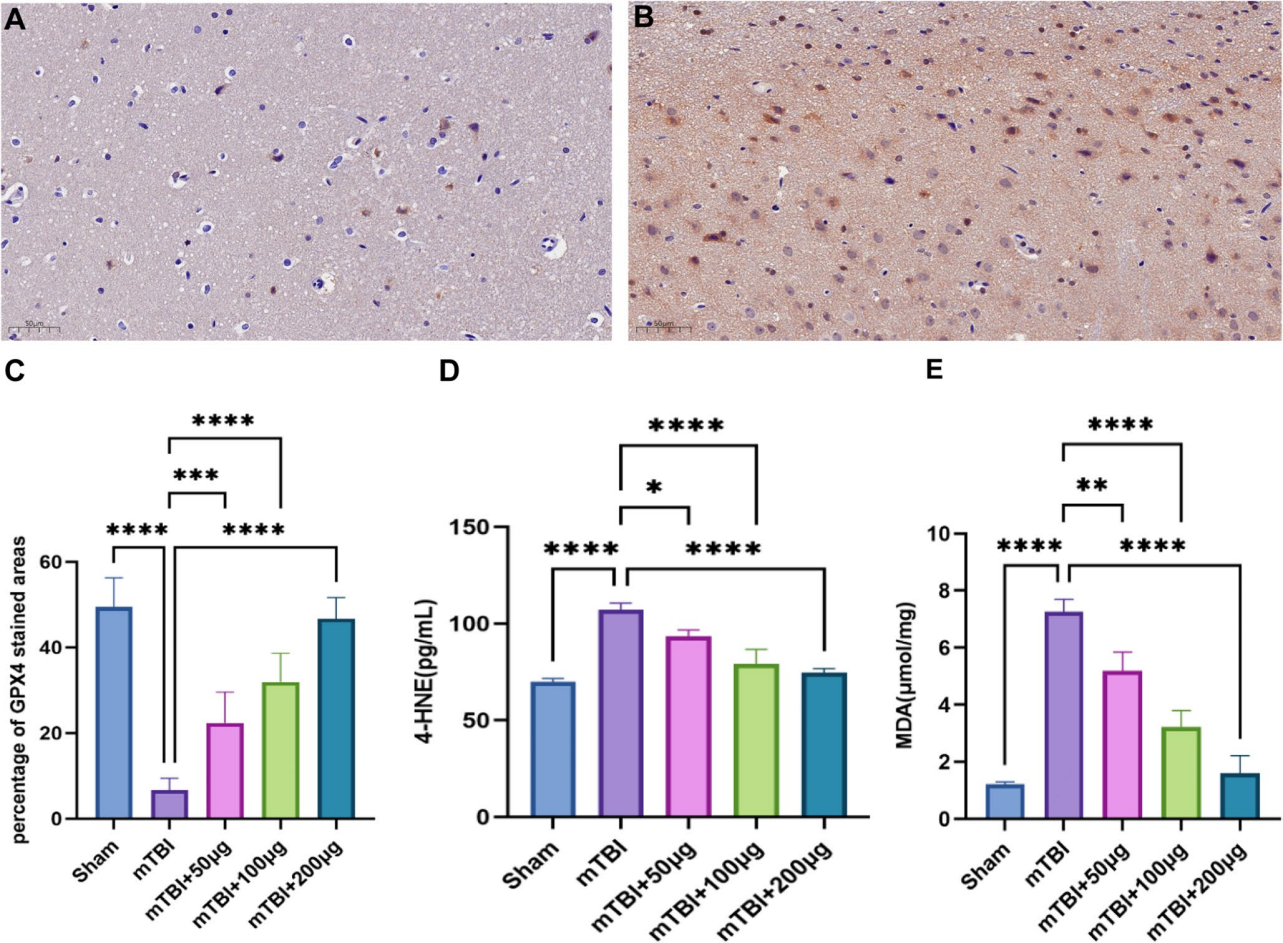
MSC-Exos restore GPX4 and reduce lipid peroxidation after mTBI. **A** GPX4 immunohistochemistry in the hippocampus of mTBI rats. **B**GPX4immunohistochemistry in the hippocampus of mTBI rats treated with 200 µg of MSC-Exos. **C** Immunohistochemical analysis showed a marked reduction in GPX4-positive areas in the hippocampus of mTBI rats. MSC-Exos treatment restored GPX4 expression dose-dependently, with the 200 µg group showing near-complete recovery to sham levels. **D** 4-HNE, another marker of lipid peroxidation, was upregulated in the mTBI group and significantly. reduced by MSC-Exos treatment. **E **MDA levels were significantly elevated in the mTBI group, indicating enhanced lipid peroxidation. MSC-Exos treatment reduced MDA levels in a dose-responsive manner. Data are presented as mean ± SD. Two-tailed tests were used. One-way ANOVA with Bonferroni’s post hoc correction. n per group: Sham = 8; mTBI = 10; MSC-Exos 50 µg = 12; 100 µg = 12; 200 µg = 12

ELISA results showed that hippocampal levels of MDA (7.9 ± 0.6 µmol/mg) and 4-HNE (108.5 ± 6.2 pg/mL)in mTBI rats were significantly elevated by 427 % and 54.3%, respectively, compared to the sham group (*p* < 0.001). MSC-Exos intervention significantly attenuated lipid peroxidation in a dose-dependent manner (Fig. 4).

MDA levels in the mTBI group were markedly higher than those in the sham group (7.9 ± 0.6 vs. 1.5 ± 0.3 µmol/mg, *p*< 0.0001)indicating enhanced lipid peroxidation. MSC-Exos administration reduced MDA levels to 5 .5 ± 0.5 and 3.6 ± 0.4 µmol/mgin the 50 µg and 100 µg groups, respectively (*p* < 0.01 vs. mTBI). In the 200 µg group, MDA levels further decreased to 2.1 ± 0.3 µmol/mg (*p* < 0.0001 vs. mTBI), with no significant difference from the sham group, suggesting that high-dose MSC-Exos effectively alleviated oxidative stress.

A Similar trend was observed for 4-HNE. MSC-Exos treatment reduced 4-HNE levelsin a dose-dependent manner: 96 .4 ± 5.7 pg/mLin the 50 µg group (*p*< 0.05 vs. mTBI) 84.1 ± 6.8 pg/mLin the 100 µg group and 76.5 ± 5.9 pg/mLin the 200 µg group (both *p* < 0.0001 vs. mTBI), indicating that MSC-Exos exert an inhibitory effect on mTBI-induced lipid peroxidation.

#### Regulation of GPX4 expression

Immunohistochemical analysis revealed a significant reduction in GPX4 expression in the hippocampal tissue of mTBI rats with the GPX4-positive area decreasing from 48.6 ± 6.1%in the sham group to 7 .5 ± 2.4% in the mTBI group (*p* < 0.0001). These findings suggest that mTBI impairs the antioxidant defense system, potentially contributing to ferroptosis (Fig. 4).

After MSC-Exos treatment GPX4 expression increased dose-dependently. The GPX4-positive areaincreased to 22 .3 ± 4.5%in the 50 µg group (*p*< 0.001 vs. mTBI) 33.8 ± 5.9%in the 100 µg group and 46.2 ± 6.7%in the 200 µg group (both *p*< 0.0001 vs. mTBI). Notably GPX4 expressionin the 200 µg group approached the level observed in the sham group, indicating that high-dose MSC-Exos effectively reverse mTBI-induced GPX4 downregulation and exert robust antiferroptotic effects.

### Activation of the PI3K/AKT/mTOR signaling pathway at the cellular level

**Fig. 5:**
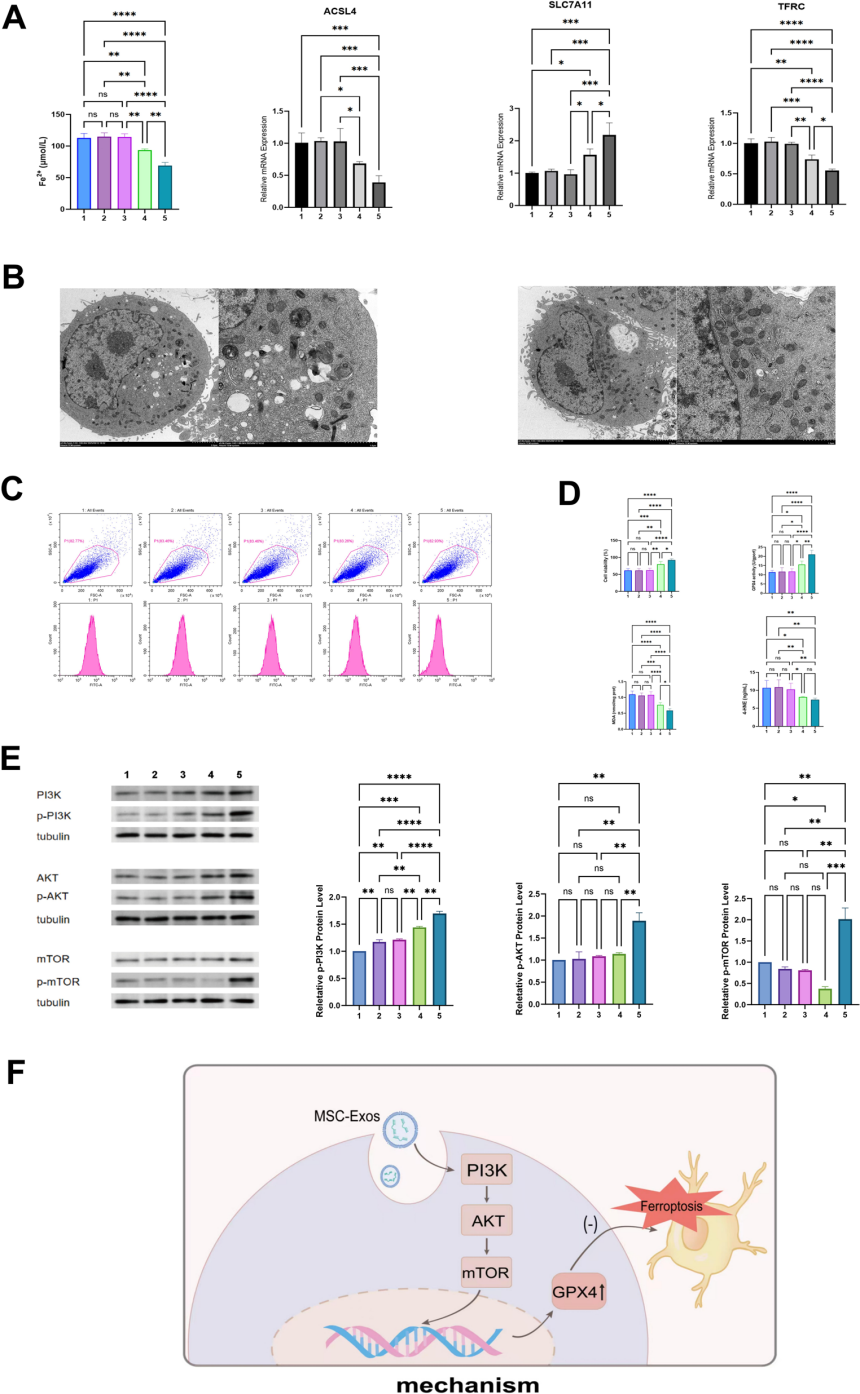
MSC-Exos suppress ferroptosis in HT22 cells through the PI3K/AKT/mTOR signaling pathway. **A** This panel shows ferroptosis data in HT22 cells. The first histogram measures the labile iron pool (LIP). It shows the amount of free iron in the cells. The next three histograms show mRNA levels of ACSL4, SLC7A11, and TFRC, which are ferroptosis-related genes. **B** TEM images of mitochondrial ultrastructure in HT22 cells. **C** Flow cytometry results show lipid ROS in HT22 cells stained with C11-BODIPY 581/591. The top plots show ROS-positive cells. The bottom histograms show fluorescence intensity. **D** Cell viability and ferroptosis-related markers were tested by CCK-8 and ELISA. **E** Western blot results show proteins of the PI3K/AKT/mTOR pathway in HT22 cells. **F **The diagram shows the mechanism. MSC-Exos go into HT22 cells and turn on the PI3K/AKT/mTOR pathway. This increases GPX4, which helps stop ferroptosis. Experimental groups were as follows: (1) Ferroptosis model (Erastin only), (2) MSC-Exos + PI3K inhibitor + ferroptosis model, (3) MSC-Exos + AKT inhibitor + ferroptosis model, (4) MSC-Exos + mTOR inhibitor + ferroptosis model，and (5) MSC-Exos + ferroptosis model. Data are presented as mean ± SD (*n* = 5 per group). Two-tailed tests were used. One-way ANOVA with Bonferroni’s post hoc correction. *p < 0.05, **p < 0.01, ***p < 0.001, ****p < 0.0001; ns: not significant

HT22 cells were used to study how MSC-Exos affect ferroptosis The ferroptosis inducer Erastin increased the level of free iron in cells. MSC-Exos reduced the LIP(*p* < 0.001). When PI3K, AKT, or mTOR inhibitors were added, this effect disappeared (Fig. 5A). The ferroptosis-related genes showed similar changes. ACSL4 went up after Erastin but went down after MSC-Exos treatment. SLC7A11 and TFRC went down after Erastin and went up after MSC-Exos. These changes were blocked by the inhibitors(Fig. 5A).

TEM showed the changes in mitochondria. In the Erastin group, mitochondria became small, dense, and lost their cristae. After MSC-Exos treatment, mitochondria looked normal and had clear cristae. The inhibitors removed this protective effect. Flow cytometry showed that Erastin increased ROS in HT22 cells. MSC-Exos reduced ROS levels. When inhibitors were added, ROS went up again(Fig. 5B).

Flow cytometry was used to measure ROS in HT22 cells stained with C11-BODIPY 581/591 (Fig. 5C). Erastin treatment caused a clear increase in ROS fluorescence compared with the control group, indicating oxidative stress during ferroptosis. MSC-Exos treatment reduced ROS accumulation and brought fluorescence intensity closer to normal levels. When PI3K, AKT, or mTOR inhibitors were added, the ROS-reducing effect of MSC-Exos was blocked, and fluorescence intensity rose again. These results show that MSC-Exos lower lipid ROS levels in HT22 cells mainly through activation of the PI3K/AKT/mTOR pathway.

Cell viability was measured by CCK-8 assay, and ferroptosis-related markers were analyzed using ELISA (Fig. 5D). Erastin treatment greatly reduced HT22 cell viability compared with the control group (*p* < 0.001). MSC-Exos pretreatment improved cell survival in a concentration-dependent way. The PI3K, AKT, and mTOR inhibitors partly blocked this protective effect.GPX4 activity was lower in the ferroptosis group than in the control group (*p* < 0.01). MSC-Exos significantly restored GPX4 activity, and the high-dose group showed the strongest effect (*p* < 0.001). Inhibitors reduced this increase, showing that GPX4 regulation depends on PI3K/AKT/mTOR signaling. MDA and 4-HNE levels, which reflect lipid peroxidation, were much higher in the ferroptosis group than in the control (*p* < 0.001). MSC-Exos reduced both markers, while pathway inhibitors weakened this effect. These findings suggest that MSC-Exos decrease lipid peroxidation and restore redox balance in HT22 cells exposed to ferroptosis.

Western blot analysis was used to measure the activation of the PI3K/AKT/mTOR signaling pathway in HT22 cells (Fig. 5E). Protein phosphorylation levels were calculated as the ratio of phosphorylated protein to total protein. Erastin treatment greatly decreased p-PI3K, p-AKT, and p-mTOR levels compared with the control group (*p* < 0.01). MSC-Exos treatment restored the phosphorylation of all three proteins in a dose-dependent way. The high-dose group showed the strongest activation of pathway proteins (*p* < 0.001). When PI3K, AKT, or mTOR inhibitors were added, the phosphorylation increase was blocked, and protein activation dropped again. These findings show that MSC-Exos promote PI3K/AKT/mTOR pathway activation in ferroptosis HT22 cells through phosphorylation-dependent regulation.

## Discussion

This study systematically investigated the therapeutic effects and underlying mechanisms of MSC-Exos in a rat model of mTBI, with a particular focus on ferroptosis and its modulation. Using a combination of behavioral assays, biochemical analyses, transcriptomic profiling, and protein validation, we showed that MSC-Exos effectively ameliorate mTBI-induced cognitive deficits. Specifically, we observed a significant increase in hippocampal lipid peroxidation markers (MDA and 4-HNE) accompanied by marked downregulation of GPX4, a key antioxidant enzyme that inhibits ferroptosis. These findings are consistent with previous studies of robust ferroptosis activation in TBI models (Wang et al. 2020), (Omelchenko et al. 2019), (Arneson et al. 2022). Moreover, the observed GPX4 downregulation aligns with the classical ferroptosis mechanism involving inactivation of the System xc⁻/GSH/GPX4 axis, suggesting that ferroptosis after mTBI is closely associated with disruption of this antioxidant defense pathway (Kumari 2025; Jia et al. 2023). Importantly, by identifying and validating the MSC-Exos/PI3K/AKT/mTOR/GPX4 axis as a novel regulatory mechanism, this study provides new insights into the molecular basis of mTBI-induced cognitive dysfunction and offers a promising therapeutic strategy for its intervention.

In this study, behavioral assessments in the animal model provided compelling evidence that MSC-Exos exert significant therapeutic effects in alleviating mTBI-induced cognitive deficits. Previous studies have demonstrated that MSC-Exos, as critical mediators of intercellular communication, carry diverse bioactive molecules, including proteins, lipids, and nucleic acids that enable them to play pivotal roles in neuroprotection and repair (Huang et al. 2024; i et al. 2022; Zhang et al. 2025). Consistent with this, MSC-Exos have been shown to markedly attenuate acute inflammatory responses, reduce brain edema and contusion volume, and improve both sensorimotor function and cognitive performance in moderate fluid percussion injury models (Blaya et al. 2025). These findings suggest that MSC-Exos may improve mTBI-related cognitive impairment by regulating neuroinflammation and promoting neural regeneration. Moreover, accumulating evidence indicates that exosome-based therapies can also confer neuroprotection through modulation of autophagic flux. For example, EPPS treatment in murine TBI models significantly reduced Aβ deposition, improved neuronal autophagy, and thereby alleviated synaptic damage and cognitive deficits (Anthony Jalin et al. 2019). By analogy, MSC-Exos may employ similar mechanisms to regulate intracellular autophagy, thereby promoting neuronal survival and functional recovery. The present findings are in line with those reported by Nie et al. in models of central nervous system disorders, further supporting the therapeutic potential of MSC-Exos in neurodegenerative disease (Nie et al. 2023). Notablyin our study, animals treated with the 200 µg dose of MSC-Exos exhibited near-complete restoration of cognitive performance. This striking recovery highlights the robust neuroprotective efficacy of MSC-Exos and suggests that their therapeutic benefits may involve multiple complementary mechanisms acting in concert to promote cognitive restoration.

To investigate the activation of ferroptosis in mTBI we assessed lipid peroxidation markers and the key regulatory protein GPX4. Our results showed significant accumulation of MDA and 4-HNE in the hippocampus accompanied by marked downregulation of GPX4indicating robust ferroptosis activation. Previous studies have reported that the accumulation of lipid peroxidation products such as MDA and 4 -HNE after TBI is closely associated with reduced GPX4 expression, highlighting the pivotal role of GPX4 in regulating ferroptosis (Kenny et al. 2019). Moreover, inhibition of ferroptosis has been shown to significantly improve neurological function after TBI, further supporting GPX4 as a potential therapeutic target (Gao et al. 2021). In the context of mTBI, both acute and chronic stress can exacerbate ferroptosis by promoting iron overload, whereas treatment with iron chelators such as deferoxamine can reverse this effect (Zheng et al. 2025). This stress–ferroptosis interaction underscores the importance of ferroptosis in the pathophysiology of mTBI. Nevertheless, despite the recognized mechanistic relevance, direct biochemical validation of ferroptosis markers and experimental evidence directly linking ferroptosis to cognitive impairment, particularly in mTBI has remained limited. Our study fills this gap by providing clear validation of lipid peroxidation dynamics and establishing a strong association between ferroptosis activation and mTBI-induced cognitive deficits. Furthermore, we demonstrated that MSC-Exos dose-dependently reduced lipid peroxidation and restored GPX4 expression, suggesting their potential to attenuate ferroptosis-driven neuronal injury and supporting their promise as a therapeutic strategy in mTBI.。.

To further elucidate the upstream regulatory mechanisms of GPX4 and ferroptosis we performed transcriptomic and bioinformatic analyses. Differential gene expression profiling revealed significant enrichment of ferroptosis-related pathways particularly the PI3K/AKT and mTOR signaling cascades which are well-established mediators of cell survival in neural injury. WGCNA further identified a gene module closely associated with cognitive outcomes highlighting GPX4 AKT1 and mTOR as key nodes within the protein–protein interaction network. Consistent with the transcriptomic findings Western blot analysis demonstrated that phosphorylation levels of PI3K (Tyr199) AKT (Ser473) and mTOR (Ser2448) were markedly reducedin mTBI rats, but were restored in a dose-dependent manner following MSC-Exos treatment. Notably, MSC-Exos administration increased phosphorylation of these proteins by 1 .45-, 1.17-, and 1.35-fold, respectively, indicating effective reactivation of this signaling pathway. These results suggest that MSC-Exos enhance GPX4 expression by activating the PI3K/AKT/mTOR cascade, thereby contributing to the suppression of ferroptosis. Previous studies have linked PI3K/AKT signaling to both neural repair and ferroptosis regulation (Chen et al. 2021). As a central regulator of cell growth, survival, metabolism, and apoptosis, the PI3K/AKT/mTOR pathway plays a pivotal role in the nervous system. Substantial evidence demonstrates that this pathway not only promotes neuronal growth and survival but also modulates multiple forms of regulated cell death, including ferroptosis. In terms of neural repair, PTEN inhibition has been shown to activate the PI3K/AKT/mTOR pathway, thereby enhancing axonal outgrowth and neuronal survival, underscoring its essential role in regeneration (Liu et al. 2019). Similarly, Homer1a confers neuroprotection against traumatic injury via modulation of PI3K/AKT/mTOR signaling (Wang et al. 2020). Regarding ferroptosis regulation, activation of this signaling cascade suppresses lipid peroxidation and attenuates oxidative stress–induced neuronal damage. For example, CISD2 protects neurons after intracerebral hemorrhage by inhibiting ferroptosis through the AKT/mTOR pathway (Li et al. 2023). Likewise, natural compounds such as vitamin E alleviate oxidative stress–induced damage in mesenchymal stem cells by modulating PI3K/AKT/mTOR signaling (Lan et al. 2022). Furthermore, this pathway is closely linked to autophagy, a process intricately associated with neuronal survival and death (Zhu et al. 2016; Hwang et al. 2017).Building upon these insights, our study extends current knowledge by directly demonstrating the association between PI3K/AKT/mTOR signaling and GPX4 regulation in mTBI, and further emphasizes the role of mTOR as a central regulatory node within the ferroptosis signaling network.

This study proposes and supports a potential MSC-Exos/PI3K/AKT/mTOR/GPX4 regulatory axis in mTBI, consistent with and extending prior exosome and ferroptosis-related work. MSC-Exos restore GPX4 expression, suppress ferroptosis, and mitigate cognitive impairment. This integrated mechanism deepens our understanding of ferroptosis regulation in mTBI and highlights MSC-Exos as a therapeutic agent capable of modulating key neuronal survival pathways. Compared with traditional stem cell therapies, MSC-Exos offer several advantages, including lower immunogenicity, superior biosafety, and enhanced blood–brain barrier permeability (Williams et al. 2020; Xu et al. 2022; Alshahrani 2024). The observed dose-dependent effects also provide experimental evidence to guide clinical dosing optimization. Notably no significant adverse effects were observedin the 200 µg treatment group, which presented the most pronounced cognitive improvement. These findings lay a strong foundation for future clinical translation and trial design, underscoring the therapeutic potential of MSC-Exos in targeting ferroptosis-mediated neurodegeneration in mTBI.

This study also has important limits that guide next steps. Species differences affect translation. Rats and humans differ in tissue complexity, neuroplasticity, and blood–brain barrier function. Nonhuman primate studies will increase clinical relevance. Sex is a biological variable. We studied only males. Female rodents may show different ferroptosis sensitivity and repair capacity (Schober 2025; Lê 2025).. Future work will include females and prespecified sex-by-treatment analyses. In line with previous studies demonstrating that MSC-Exos are capable of crossing the BBB, we did not track these vesicles in vivo in the present study (Xiao et al. 2025; Ma et al. 2024). Future experiments involving labeled exosomes and tissue quantification will be conducted to assess BBB crossing, regional retention, and cellular uptake of MSC-Exos. We focused on the PI3K/AKT/mTOR/GPX4 axis. We did not compare this axis with other ferroptosis-regulatory pathways, such as Nrf2/HO-1 and FSP1/CoQ10. Head-to-head comparators, including standard ferroptosis inhibitors, will help define relative therapeutic value. Pharmacological inhibitors supported pathway dependence, but genetic tools will strengthen causality. GPX4 gain- and loss-of-function and targeted editing of PI3K/AKT/mTOR can test necessity and sufficiency. Manufacturing at GMP scale, long-term safety, and dose translation also require study. Pharmacokinetic and pharmacodynamic analyses will define exposure, brain reach, and optimal schedules.

In conclusion, this study provides systematic evidence that ferroptosis contributes to mTBI-related cognitive dysfunction. We also propose and support a potential MSC-Exos→PI3K/AKT/mTOR→GPX4 axis that may mediate these effects. This mechanism offers a clear biological framework and a testable therapeutic target for cognitive deficits after mTBI. Our findings further suggest that MSC-Exos have translational promise. Future work should define optimal dosing, delivery, and safety in clinically relevant models.

## Data Availability

Due to the ongoing nature of follow-up studies based on these data, the datasets generated and analyzed during the current study are not publicly available at this time. Interested researchers may contact the corresponding author to request access under reasonable conditions.
